# Health professional assisted Kangaroo mother care practice in Ethiopian health care facilities: evidence from the 2016 Ethiopian demographic and health survey

**DOI:** 10.1186/s12887-023-04230-8

**Published:** 2023-08-24

**Authors:** Mesfin Wudu Kassaw, Ayele Mamo Abebe, Biruk Beletew Abate, Ayelign Mengesha Kassie, Kirubel Dagnaw Tegegne

**Affiliations:** 1 Department of Nursing, College of Health Science, Woldia University, Woldia, Ethiopia; 2 Department of Nursing, College of Health Science, Debre Berhan University, Debre Berhan, Ethiopia; 3 Department of Nursing, College of Health Science, Wollo University, Dessie, Ethiopia

**Keywords:** KMC, Health Professional, Health Facilities, 2016EDHS, Health Facilities, Mothers

## Abstract

**Background:**

Worldwide, 15 million children born prematurely every year and over one million of them died because of prematurity caused complications. However, three-fourths of deaths from preterm related complications are preventable by using Kangaroo Mother Care (KMC). The Ethiopian government has been implementing a guideline that declares putting all low birth weight neonates at KMC. The aim of this study was to assess health professionals’ assisted KMC practice and its associated factors among Ethiopian mothers who gave birth at health facilities.

**Methodology:**

This study used the 2016Ethiopian Demographic and Health Survey data (EDHS). The 2016EDHS used a stratified two stage sampling method to select a representative sample using validated questioner. The sample we used in this study after cleaning the children’s data set from the 2016EDHS was 2,960. Logistic regression model was used to assess the association of health professional assisted KMC practice and predictor variables.

**Results:**

Mothers who gave birth in health facilities and practiced kangaroo mother care were 1808(62.1%). In the multivariable logistic regression analysis, women from poorest (AOR, (95%CI)), (0.60, (0.43, 0.81)) and poorer (0.62, (0.46, 0.86)) socio-economic status were not practicing KMC.

**Conclusions:**

The coverage of health professional assisted KMC practice was far lower than the expectation for mothers who gave birth in health facilities (100%). Low socio-economic status was associated with not practicing KMC. A further study on why mothers from low wealth index did not practicing KMC while they were in health facilities may be needed.

## Background

Worldwide, 15 million children born prematurely every year and over one million died because of prematurity caused complications [1]. Pre-term birth is the direct and leading cause to three million neonatal deaths each year and the second leading cause of all deaths in under-five children globally [2, 3]. However, three-fourths of deaths from preterm related complications are preventable without intensive care units [4] using KMC. Since neonatal period by itself presents the greatest risk of death, which is exacerbated for preterm newborns as they have less physiological reserve, have greater challenges in temperature regulation, have immature organs, have poor immune function, and heightened vulnerability to severe infections. These all physiologic characteristics of preterm newborns putting them at risk for problems associated with the transition to extra uterine life [5, 6]. Fortunately, neonatal health begun to emerge as a global and national public health priority especially through attention to child survival in the Millennium Development Goals [7], which contribute more to emphasize on Kangaroo Mother Care (KMC). Kangaroo Mother Care is a method of holding a neonate in skin-to skin contact (STS) at prone and upright position on the maternal chest in which the neonate is enclosed in maternal clothing for thermal regulation [8]. KMC is an evidence-based inpatient care technique for premature and low-birth weight (LBW) neonate when it weighs less than 2000gm [9, 10]. It is an easily available and biologically sound method of care for all newborns, but in particular for premature newborns [11]. The clinical efficacy and health benefits of KMC to preterm and low-birth weight neonates (< 2000gm) has been widely demonstrated. According to a study, KMC can prevent up to half of all deaths in neonates weighing < 2000gm at birth [10]. However, global KMC coverage has been remained low, and strategies to scale up KMC uptake have not been effective. Researchers indicated barriers of KMC uptake including high staff turnover, lack of KMC services in health facilities, and omission of KMC indicators in health management information systems [12, 13] globally and in Ethiopia also. But, KMC as a means of care for preterm infants in health facilities was ranked second out of 82 approaches in global research priority setting for newborn babies, which is really indicative about the importance of KMC implementation [14]. In comparing to Ethiopia, KMC has been widely implemented in western and some African countries including Nigeria, Madagascar, Malawi, Ghana, Indonesia [15, 16] and South Africa [17].

Following the concern given to newborn health in the agenda of millennium development goal, both the Ethiopian minister of health and World Health Organization (WHO) guidelines included and stated that all LBW babies need to receive KMC. However, sick and very small neonates should initially be cared in a radiant warmer and KMC would be initiated once the baby is thermodynamically stable [12]. Despite the initiative taken by Ethiopian Minister of Health, a study in Ethiopia reported the presence of poor KMC infrastructure, low KMC initiation and poor survival among those who received KMC. The study was focused mainly on low birth weight babies and the initiation of KMC was 46.4% only [18]. By considering other options of LBW management like radiant warmer, we are interested in overall KMC coverage among mothers who gave birth in health facilities. Taking the advantage that KMC can be used in economically challenged countries like Ethiopia where access to incubators is limited or too expensive for the whole population to each end of periphery [19], Ethiopia start using KMC as a strategy to decrease neonatal mortality as it was stated clearly in the health sector transformation plan (HSTP), but only 46.4% of eligible neonates were received KMC [18] in the beginning years. The coverage of KMC in the country has remained low and its implementation has largely been limited to specialized hospital that are located in big cities of Ethiopia [20, 21] in the consecutive early years since it started. However, KMC shorten hospital stay and create opportunities for teaching mothers and assessing the progress of the neonate, and open a means for better use of health care service [10, 22–24]. A study also indicated the existence of difference in between health facilities on practicing KMC in Ethiopia [25]. Thus, the aim of this study was to assess health professional assisted Kangaroo mother care practice and its associated factors in mothers who gave birth in health facilities in Ethiopia.

## Methodology

### Data collection period, and study population

The data collection period for the 2016EDHS was from January 18 to June 27, 2016. The 2016 Ethiopian Demographic and Health Survey (EDHS) data was used for this study. The 2016 EDHS data was the fourth survey conducted in Ethiopia. The survey collected information on household’s and respondent’s characteristics, child health, infant and child mortality, malaria, maternal health, maternal mortality, nutrition, tobacco use, women’s empowerment, anemia, domestic violence, environmental health, family planning, fertility and fertility preferences, and etc. using validated questioner. The detail about all the surveys of EDHS including questioner and their code is available at dhs.com. The purpose of the EDHS is to provide up-to-date estimates of the key demographic and health indicators of the population [26]. The survey included reproductive age group women, under-five children, and productive age group men (15-59years old) [26, 27].

### Sampling techniques, procedures and study design

The 2016EDHS data was collected using a stratified two stage sampling method to select a representative sample. All the regions of the country were stratified into urban and rural areas. From all the 11administrative states, 21sampling strata were yielded. The samples of enumeration areas (EAs) were selected independently in each stratum.

The explicit stratification and proportional allocation were achieved at each of the lower administrative levels by sorting the sampling frame within each sampling stratum before sample selection according to the administrative units in the different levels, and by using a probability proportion to size selection at the first stage of sampling. For the 2016EDHS data collection, 645 EAs were selected with a probability proportional to the EA size and with independent selection in each sampling stratum. The EA size is the number of residential households in the EA that was determined in the 2007 Ethiopian Population and Housing Census. According to the 2016 EDHS procedures, a household listing operation was implemented in the selected EAs, and the resulting lists of households served as the sampling frame for the selection of households in the second stage. The data collectors interviewed only the pre-selected households. In the EDHS, there were no replacements or changes of the pre-selected households in the implementing stages to prevent bias. All the under-five children, who were members of the selected households or who spent the night before the survey in the selected households were eligible for the child survey [27].

### Data collection

The 2016EDHS usually use five groups of questionnaires in collecting the data. Those questionnaires are the household questionnaire, the woman’s questionnaire, the man’s questionnaire, the biomarker questionnaire, and the health facility questionnaire. The questionnaires were adapted from the DHS program’s standard demographic and health survey questionnaires in a way to reflect the population and health issues relevant to the Ethiopian context. Questions that stated about children were integrated to woman’s questionnaire. The outcome variable of this study is health professional assisted KMC. The independent variables were socio-demographic variables of both children and mothers, health services provided to children and the wider community, and substance use like cigarette smoking and Khat chewing in considering the availability of the variables in the 2016EDHS data [26, 27].

### Measurement of variables

#### KMC

Holding a neonate in the mothers’ skin in a prone and upright position on the maternal chest that also enclosed in maternal clothing for thermal regulation [8]. This is a practice of putting the neonate in the mothers’ chest under her cloth and initiate breast feeding in KMC room predominantly arranged for KMC service.

#### Health professional assisted KMC

Practice of KMC by mothers under the health care professionals (HCPs) advice given for mothers who gave birth in health facilities. The women might heard or aware about KMC before birth, even during birth and after birth through professional patient commination.

#### Wealth index

EDHS data usually grouped socio-economic class of the community in to five; poorest, poor, medium, rich, and richest. This wealth index classification is based on the property of the household, which are different for the urban and rural areas. The questionnaires for the rural and urban areas and the approach to compose all questionnaires in to one score is described in 2016EDHS coding document, which is available at dhs.com.

#### Child size at birth

The weight of newborns in EDHS is not measured objectively by health professionals. It was collected by asking mothers whether the approximate size of their newborn was very large, large, average, small or very small.

### Data analysis

SPSS version 23 has been used to analyze the data. The data analysis started with a summary of socio-demographic characteristics, and other relevant factors of the respondents using descriptive statistics. Before analyzing the data, weighting, cluster and strata adjustment were performed to get representative sample and appropriate estimate relative to population sizes. Logistic regression model was used to assess the association of health professionals assisted KMC with predictor variables. Variables with a p-value of 0.25 and below in the crude association were included in to the adjusted model. A statistically significant association was determined at a p-value of less than 0.05 in the final adjusted model.

## Results

### Socio-demographic characteristics of study subjects

The 2016EDHS child data set has 10641observations. After excluding children who were not born in health facilities and observations with missing values, 2910 mothers and under-five children pairs were included for this further secondary data analysis.

Of the total participants, 1040(35.7%) husbands had agricultural jobs, and 1658(57.0%) mothers were housewife. More than 57% 1667(57.3%) of mothers were live in the rural areas, 2867(98.5%) mothers were married, and 1036(35.6%) mothers were completed primary education. Regarding religion, 1224(42.1%) mothers were affiliated to Ethiopian Christian Orthodox, and 1139(39.1%) mother were unable to read and write (Table 1).

**Table 1:** Socio-demographic characteristics of mothers and their children in Ethiopia, (n = 2910)

Variables	Categories	Frequency(n)	(%)
Paternal occupation	Do not work	398	13.7
	Professional/managerial	355	12.2
	Clerical	37	1.3
	Sales	397	13.6
	Agricultural employee	1040	35.7
	Service	181	6.2
	Skilled manual	356	12.2
	Unskilled manual	146	5.0
Maternal occupation	Do not work	1658	57.0
	Professional/managerial	118	4.1
	Clerical	38	1.3
	Sales	446	15.3
	Agricultural employee	424	14.6
	Service	67	2.3
	Skilled manual	112	3.8
	Unskilled manual	47	1.6
Head of household	Male	2350	80.8
	Female	560	19.2
Maternal residence	Urban	1243	42.7
	Rural	1667	57.3
Maternal age	15–19	143	4.9
	20–24	718	24.7
	25–29	894	30.7
	30–34	604	20.8
	35–39	401	13.8
	40–44	124	4.3
	45–49	26	0.9
Maternal education	No education	1139	39.1
	Primary	1036	35.6
	Secondary	453	15.6
	Higher	282	9.7
Region	Tigray	484	16.6
	Afar	111	3.8
	Amhara	230	7.9
	Oromia	267	9.2
	Somali	237	8.1
	Benishangul	213	7.3
	SNNPR	322	11.1
	Gambela	199	6.8
	Harari	265	9.1
	Addis Adaba	334	11.5
	Dire-dawa	248	8.5
Religion	Orthodox	1224	42.1
	Catholic	18	0.6
	Protestant	443	15.2
	Muslim	1206	41.4
	Traditional	6	0.2
	Other	13	0.4
Wealth index	Poorest	436	15.0
	Poorer	393	13.5
	Middle	362	12.4
	Richer	397	13.6
	Richest	1322	45.4
Readiness to have child	Wanted then	2315	79.6
	Wanted later	452	15.5
	Wanted no more	143	4.9
Cigarette smoking	No	2886	99.2
	Yes	24	0.8
Chat chewing	No	2600	89.3
	Yes	310	10.7
Type of birth	Single birth	2814	96.7
	1st of multiple birth(twins)	51	1.8
	2nd of multiple birth(twins)	45	1.5
Sex of the child	Male	1477	50.8
	Female	1433	49.2
Marital status	Married	2867	98.5
	Living with a partner	43	1.5
Child size at birth	Very large	552	19.0
	Large	446	15.3
	Average	1277	43.9
	Very small	227	7.8
	Small	408	14.0

### Health professional assisted KMC

**Fig. 1:**
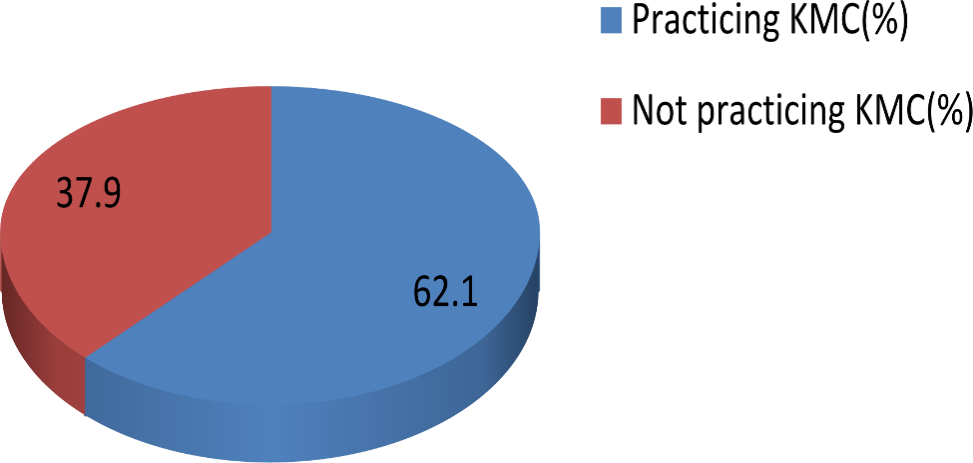
Health professional assisted kangaroo mother care practice in mothers who gave birth at health facilities in Ethiopia

The coverage of health professional assisted KMC practice among mothers who gave birth in a health facilities were only 1808(62.1%),95%CI (60.3, 63.9) despite the place that all women gave birth in a health facilities under the support of trained health professionals(Fig. 1).

### Factors associated with health professionals assisted KMC

On the crude logistic regression analysis; wealth index, region, cigarette smoking, religion, maternal education and child size were associated with health professional assisted KMC practice. But in the final adjusted multivariable logistic regression model; only women with poorest (AOR, (95%CI)), (0.60, (0.43, 0.81)), and poorer (0.62, (0.46, 0.86)) wealth index were had association with not practicing KMC (Table 2).

**Table 2:** The association of health professionals assisted KMC practice with predictor variables in Ethiopia (n = 2910)

Variables	Categories	HCPs assisted KMC	OR	OR		95% CI		P-
		No, n (%)	Yes, n (%)	COR	AOR	Lower	Upper	value
Wealth index	Poorest	216 (19.6)	220(12.2)	0.47*	0.6	0.43	0.81	0.001
	Poorer	189 (17.2)	204(11.3)	0.50*	0.62	0.46	0.86	0.001
	Medium	138 (12.5)	224(12.4)	0.76*	0.95	0.68	1.32	0.76
	Richer	139 (12.6)	258(14.3)	0.86	1.06	0.78	1.45	0.72
	Richest	420 (38.1)	902(49.9)	1	1			
Residence	Rural	712 (64.6)	955(52.8)	0.61*	0.77	0.59	1	0.05
	Urban	390 (35.4)	853(47.2)	1	1			
Household head	Male	896 (81.3)	1454(80.4)	1	1			
	Female	206 (18.7)	354(19.6)	1.06	1.03	0.84	1.25	0.8
Khat chewing	No	977 (88.7)	1623(89.8)	1	1			
	Yes	125 (11.3)	185(10.2)	0.89	0.86	0.67	1.1	0.23
Child sex	Male	576 (52.3)	901(49.8)	1	1			
	Female	526 (47.7)	907(50.2)	1.1	1.06	0.91	1.24	0.43
Maternal	Not educated	480 (43.6)	659(36.4)	0.70*	1.07	0.79	1.45	0.68
education	Primary	375 (34.0)	661(36.6)	0.9	1.17	0.87	1.57	0.29
	Secondary	152 (13.8)	301(16.6)	1.01	1.11	0.81	1.53	0.52
	Higher	95 (8.6)	187(10.3)	1	1			
Child size at birth	Very large	223 (20.2)	329(18.2)	1	1			
	Large	162 (14.7)	284(15.7)	1.19	1.16	0.89	1.5	0.28
	Average	451 (40.9)	826(45.7)	1.24*	1.22	0.99	1.51	0.06
	Small	93 (8.4)	134 (7.4)	0.98	1.03	0.75	1.42	0.85
	Very small	173 (15.7)	235(13.0)	0.92	0.96	0.74	1.25	0.76
ARDS	No	991 (89.9)	1642(90.8)	1	1			
	Yes	111 (10.1)	166 (9.2)	1.11	1.03	0.81	1.03	0.85

*p-value < 0.05 for the crude association, ARDS-acute respiratory distress syndrome, KMC- kangaroo mother care, HCP- health care professional

## Discussions

Previous evidence indicated that KMC practice for at least one hour has been improved exclusive breast feeding, maintains body temperature to normal, and keeps newborn vital signs within the normal range [28, 29]. The practice of KMC in mothers who gave birth in the health facilities was 1808 (62.1%), 95% CI (60.3, 63.9). The remain1102 (37.9%), 95%CI (36.1, 39.7) mothers did not practice KMC though they gave birth at health facilities. The current study’s coverage of KMC practice was higher than studies that reported 41.9%KMC(community-based) [30] in Yirgalem Town(Ethiopia), 46.4% KMC and 54.15% KMC(hospital-based) [31] in Lagos University Teaching Hospital, Idi-Araba (Nigeria), and 28.1% KMC(health facility based) [32] in four big cities of Ethiopia. The difference might be due to study population difference, sample size difference, inclusion and exclusion criteria, and socio-demographic difference. This current study considered only mothers who followed by health professionals in all before birth, during birth, and post-partum phases. However, all the referred papers included mothers who visited health facilities in the post-partum period irrespective of their place of birth. The present study reported a lower KMC practice than a study reported from Ghana that was 84.6% [33]. This variation might be because of socio-demographic difference between Ghana and Ethiopia. In addition, the health care service provided in Ghana might be better than in Ethiopia. Furthermore Ethiopia integrated KMC to the National Strategy for Newborn and Child Survival lately in 2015/16 than Ghana that started some years before Ethiopia [34]. The current study have a similar KMC practice with a study that conducted from Ghana, home based KMC practice [33], and other study that reported 61.6% [35]. This also reflects the socio-demographic difference between the study areas, Ethiopia and Ghana. Because the KMC practice in the Ethiopian health facilities become equal with Ghanaians Home based KMC practice. In assessing the association of health professional assisted KMC practice and its associated factors; religion, residence, wealth index, maternal education, and birth weight were associated with health professional assisted KMC practice at crude logistic regression model. However, in the final adjusted multivariable logistic regression model; only wealth index was associated with health professional assisted KMC practice. Mothers in the poorest (AOR, (95%CI), (0.60, (0.43, 0.81) and poorer (AOR, (95%CI), (0.62, (0.46, 0.86) wealth index class were not practicing KMC than mothers from richest households. However, a study from Aksum, Ethiopia reported the lack of association between KMC practice and wealth index [36]. The difference in these studies might be as a result of study area variation. This current study derived from the 2016EDHS, in which both urban and rural areas were represented proportionally. However, the study from Aksum considers only urban dwellers that might have similar degree of economic class and awareness about KMC. In addition, the study from Aksum might be designed properly to measure KMC precisely compared to the 2016EDHS. However, a qualitative study clearly indicated that socioeconomic class is the major barrier of KMC practice [13]. In this study, maternal education was not associated with practicing KMC. The reason might be because of the widely held community beliefs about KMC, which makes educated and non-educated women equal in practicing KMC as explained by Selamawit et al. in their qualitative study [13]. The average birth size newborn was 1.22(0.99, 1.51) times marginally likely to get KMC than very large newborns. However, it is not significant at 5% and needs further study to conclude whether average size babies were receiving KMC care compared to very large babies. Similarly, residence is marginally associated with KMC practice. Mothers from rural areas are less likely to practice KMC by 27% (0.77(0.599, 1.00)) compared to women in urban areas. However, this must be also taken seriously like the average birth size reported above. The reason for having marginal association of both residence and maternal education with KMC might be because of the sample size or other uncontrolled variability in the analysis or uncollected confounders. The implication of this study for researchers and policy personnel would be the wealth index and rural areas, which are the predictors of not practicing KMC. Most Ethiopian is living in rural areas and is in the lowest wealth index class. Thus, it would be good to give priority for rural areas to increase coverage of KMC and decrease neonatal mortality to achieve sustainable development goal. However, this study is not high quality in data collection or analysis to forward strong evidence, and thus we would recommend a prospective cohort study to confirm the non-significant as well the significant variables of this secondary analysis.

## Conclusions

The prevalence of health professional assisted KMC practice was far lower than the expectation for mothers who gave birth in health facilities (100%). Low socio-economic status (low wealth index) was associated negatively with KMC (not practicing) while higher wealth index was associated positively with KMC (practicing). The reason might be that mothers from higher wealth index households might have also higher awareness about KMC. A further study on why mothers from low wealth index did not practicing KMC while they were in health facilities may be needed.

### Limitations

This study has four limitations. The first limitation is exclusion of variables because of missing values. And due to the exclusion of observations, the sample size was small that might compromise the precision of this research. The second limitation is the survey didn’t include all the important clinical variables that have better probability to predict KMC practice. The third limitation might be the recall bias. The women asked about KMC practice after years of birth and they might not really remember what they did in the hospital. The last limitation might be the nature and goal of the EDHS might not be a right data source to model association studies because EDHS is interested in describing demography of the population and the tools are designed to answers this goal.

## Data Availability

The data that support the conclusions of this research is already online in the DHS database online at https://www.dhsprogram.com/.

## Abbreviations

AOR: adjusted odds ratio
CI: confidence interval
SPSS: statistical package for social science
WHO: World Health Organization
EDHS: Ethiopian demography and health survey
DHS: Demography and health survey
SNNPR: Southern nation nationalities and people representatives
EA: Enumeration areas
USAID: The United states Agency for International Development
ICF: Inner City Fund
KMC: Kangaroo Mother Care
LBW: low-birth weight
NMR: newborn mortality
